# Prevalence of Hypertension and its Associated Factors among Public Vehicle Drivers in Western Nepal: A Population-based Study

**DOI:** 10.21203/rs.3.rs-6310909/v1

**Published:** 2025-06-09

**Authors:** Nawaraj Chapagain, Shivaraj Mishra, Barun Kumar Singh, Tulsiram Bhandari, Baburam Acharya, Nand Ram Gahatraj

**Affiliations:** Nutrition Research Project of Kansai Medical University Japan in Annapurna Rural Municipality; The University of Sydney; Nepal One Health Institute; Pokhara University; Deakin University; Pokhara University

**Keywords:** Drivers, High Blood Pressure, Hypertension, Prevalence, Nepal

## Abstract

**Introduction:**

Hypertension presents a major global health problem, impacting persons across all ages, backgrounds, and lifestyles. Occupational risk factors interlinked to hypertension are poorly researched in Nepal. The study aimed to measure the prevalence of hypertension and its associated factors among public vehicle drivers.

**Methods:**

In this cross-sectional study, we enrolled 358 public vehicle drivers from Pokhara Metropolitan City by visiting randomly selected parking stations and approaching divers during their rest time. Trained examiners measured blood pressure for three times and completed a questionnaire to assess demographic, lifestyle and occupational factors. Blood pressure of 140/90 or more was diagnosed as hypertension. The chi-square test and binary logistic regression were used to identify the risk factors of hypertension.

**Results:**

The mean age of respondents was 37.3 ± 8.5 years, and all of them were male. The prevalence of hypertension was 41.9%. Age [adjusted odds ratio (aOR)=1.84], BMI (aOR=0.46), alcohol (aOR 0.44), and medication (aOR=0.05) were found to be significantly associated with hypertension.

**Conclusion:**

Nearly half of drivers had hypertension, which was associated with age, being overweight, alcohol and substance use and family history of hypertension. Drivers with these particular risk factors should be targeted for screening and management of hypertension.

## Background

Hypertension effects 1.28 billion people globally.^1^ Modified risk factors such as poor dietary habits (excessive sodium intake, a diet rich in saturated and trans fats, and insufficient consumption of fruits and vegetables), sedentary lifestyle, tobacco and alcohol use, and overweight or obesity play a role in increasing the risk of hypertension.^2^ Despite the significant prevalence of hypertension, knowledge, treatment, and management remain inadequate, especially in low- and middle-income countries (LMICs).^3,4^ Studies indicate that a significant number of patients with hypertension are misdiagnosed, and among those who are diagnosed, medication adherence is frequently inadequate.^5^ Limited access to healthcare facilities, financial limitations, insufficient awareness of the long-term consequences of unmanaged hypertension, and cultural attitudes inhibit effective hypertension management in these contexts.^6^ Moreover, healthcare systems in LMICs face difficulties in executing efficient screening, early detection, and follow-up methods, thereby increasing the illness burden.^7^ Strengthening public health initiatives, increasing access to affordable drugs, and encouraging lifestyle changes are critical for addressing these problems and improving hypertension control in limited-resource countries.^8^

Among various occupational groups, both short and long (bus) drivers represent a substantial workforce exposed to prolonged sitting, irregular work hours high levels of stress, and environmental factors such as air pollution and traffic-related stressors.^9^ The prevalence of hypertension has been observed to vary among different occupational categories; some studies have revealed a frequency of 26%, 21.3%, or 14.8% among hospital staff^10^, while others reported a prevalence of 34.4% and 22.2% among bankers and traffic wardens, respectively.^11^

Nepal, following the WHO Global Action Plan, has adopted the goal of achieving a 25% relative decrease in the prevalence of elevated blood pressure by 2025 compared to the levels recorded in 2010, aligning with the global targets for non-communicable diseases (NCDS) control.^12^ The etiology of hypertension is influenced by several risk variables, including age, location, genetics, socioeconomic status, ethnicity, diet, and nutrition. Among the important risk factors for hypertension are factors related to our line of work.^13^

Similarly, Nepal has made significant steps in the management of chronic diseases with the introduction of WHO-PEN, which is also recognized as an effective intervention program for NCDs^14^ that can reduce NCDs-related premature death by 25%. The Multi-Sectoral Action Plan for the Prevention and Control of Non-Communicable Diseases (2014-2020) provided the foundation for the formulation of the PEN Implementation Plan (2016-2020) in Nepal. The integration of NCDS services into Nepal’s primary health care (PHC) system was aided by the development of healthcare professionals’ capacity in the management of NCDs^15^. Currently, the prevalence of hypertension in Nepal is 24.5%.^16^ Gaps in hypertension management and regulation may arise from various factors, including health system-related barriers such as limited access, poor service delivery, and unaffordability.^17^

This is the first attempt to concurrently investigate the relationship between lifestyle, anthropometric, and sociodemographic variables and hypertension among public transportation drivers. Early identification of those who are at risk will facilitate prompt intervention and support efforts to avoid cardiovascular disease. This study aimed to ascertain the prevalence of hypertension and its associated factors among drivers of public transportation in Pokhara, Nepal.

## Methods

### Study Design, Setting, and Study Population

A cross-sectional study was conducted from April to December 2023 in Pokhara Metropolitan of Kaski, situated in central Nepal and serves as the capital of Gandaki Province. It is the second most populous city in Nepal, following Kathmandu, with a population of 599,504 residing in 120,594 houses as of 2021.^18^ The study population was drivers aged above 18 years with a government-registered license. A public vehicle driver is primarily responsible for transporting individuals and goods from one location to another using designated vehicles for public transportation, such as buses, taxis, and micro-buses.

### Sampling Procedures

The sampling procedures for our research involved three key steps. First, we identified the total number of registered drivers using data from local public transportation authorities. Second, we employed a lottery method to randomly select parking stations, where drivers were available, to ensure an unbiased selection process. Finally, we used convenience sampling at these selected stations, where data was obtained from drivers who were readily available and willing to participate, gathering anthropometric and demographic data from them.

### Data Collection Procedures

Face-to-face interviews were used to collect data from drivers for sociodemographic, behavioural, and work-related characteristics, and physical measurements. The study tool was adapted from the World Health Organization’s validated STEPS tools version 3.1^19^, which have been validated in Nepali by the Nepal Health Research Council (NHRC).^16^ For income, we used the basic level as defined by the Ministry of Labor, The Government of Nepal’s Ministry of Employment and Social Security has published a notification through the Nepal Gazette.^20^ Anthropometric measurements were carried out using established techniques and calibrated instruments.

### Blood Pressure Measurement

Participants were asked to sit after resting for at least fifteen minutes, and blood pressure was recorded using a digital measuring instrument (Omron-WHO recommended). Five minutes apart, three blood pressure readings were obtained. The study considered the mean of the previous two readings. A systolic blood pressure measurement of no less than 140 mm Hg and a diastolic blood pressure measurement of no less than 90 mm Hg, or antihypertensive medication was considered hypertension.

### Anthropometric Measurements

Weight was measured using a digital scale, and height was measured using a Height Measuring Stadiometer. Waist and hip circumferences were measured using Seca 201 Measuring tape. Body mass index (BMI) is computed by dividing weight in kilograms by height in meters squared. BMI was classified as normal weight (18.5-24.9 kg/m2), overweight (25-29.9 kg/m2), or obese (≥ 30 kg/m2).^21^ Waist circumference (WC) that is specific to males can be defined as normal (<94 cm), overweight (94-101.9 cm), and being obese (≥102 cm), whereas male waist-to-hip ratio (WHR) is classified as normal (<0.90), overweight (0.90-0.99), and obese (≥1.0). ^22^ Waist and hip circumferences have been measured with Seca 201 Measuring Tape. As all participants were male, participants with waist circumference ≥ 90 centimeters were regarded as having central obesity.^23^

### Awareness Treatment and Control of HTN

Participants who indicated that a physician never informed them of their hypertension were considered conscious of their hypertensive status. Participants who received any form of antihypertensive medication within the prior two weeks were marked as undergoing treatment.^24^ Hypertensive participants with an average systolic BP < 140 mm Hg and an average diastolic BP < 90 mm Hg were considered to be under control.^25^

### Data Collection and Measurements

The data collection tool was developed after an extensive review of related literature and prepared under continuous and close guidance and supervision of the academic supervisor. Reliability was ensured after the pretesting of the tools in a homogenous area. Tools were prepared in both English and Nepali language. A validated and WHO-recommended standard tool was used to assess the prevalence of hypertension and associated factors. The data entry format was prepared by REDCap software for entering the collected data in a precise manner. A pre-test was conducted among 20 drivers at the Dhulegauda parking station to check the quality of the tool and little modification was done. Moreover, software handling and errors were checked before the data collection, and the questionnaire was checked before deploying the form on REDCap.

The dependent variable for this study, hypertension had a yes/no dichotomy. If a participant had at least one of the following characteristics or was receiving antihypertensive medication, they were deemed to have hypertension: (SBP ≥ 140 and DBP ≥ 90 mmHg)^26^.

The independent variables for this study were age, ethnicity, marital status, degree of education, monthly income, body mass index, waist circumference, waist-height ratio, cigarette smoking, alcohol consumption, physical activity, diet, diabetes status, and family history of HTN, working experience, work hour per day (WHPD), workhour per weak (WHPW), rest break between driving, driving vehicle, Weekly average driving (WAD) and types of road.^27–29^ Considering prior academic and professional experiences, WHPD, WAD, and WHPW were assessed by classifying participants into <13 and ≥13 years of experience, <8 and ≥8 hours per day, ≤800 km and >800 km, and ≤48 and >48 hours per week respectively.^27^

### Analysis

The gathered data were checked for consistency and completeness before being entered into REDCap Software. (v5.27.2) and analyzed using IBM SPSS Statistics for Windows, version 20.0 (Armonk, NY: IBM Corp). Descriptive statistics was shown as frequency, and percentage for categorical variables and mean, SD for continuous variables. Then, the chi-square test was applied to test the association between the independent and dependent variables. Finally, binary logistic regression was used to find factors associated with hypertension among drivers. In which variables having a p-value less than 0.25 were moved into multivariable binary logistic regression. Before moving in binary logistic regression, multicollinearity was checked, through variance inflation factor (VIF).^30^ A P-value less than 0.05 was considered statistically significant in binary logistic regression.

Frequency distributions, means and percentages were calculated as descriptive analysis. A multiple logistic regression analysis was performed with hypertension as the dependent variable and age, sex, ethnicity, smoking, alcohol consumption, physical activity, BMI, central obesity, and positive family history as the independent variables. Those variables with a p-value ≤ 0.05 were considered statistically significant associations.

### Ethical Approval and Consent of Participants

This study received ethical approval from Pokhara University’s Institutional Review Committee (020-080/81, 15-09-2023). Similarly, written permission was taken from the health division of Pokhara Metropolitan City. The aim, importance, and methodology of the research were also explained to every participant. Furthermore, they were told that they have the right to withdraw from the activity at any moment and that their identity will remain anonymous while information is captured. The data-gathering procedure then started after written informed consent was received. First-time hypertensive patients were encouraged to consult a doctor during the data collection period.

## Results

### Socio-demographic Characteristics

A total of 358 were involved in the study (Table 1). The mean age of the participants was 37 years. About two-fifths (39.7%) of the participants were of the age group 36-45 years. More than two-fifths (42.5%) of participants were Janajati. Almost all (92.7%) participants were married. Then, half of the participants had completed a secondary level of education. Almost all (96.6%) of the participants had monthly income as per Nepal Rastra Bank. Furthermore, a family history of hypertension was present in three-fifths (61.7%) of the patients. Nearly one-fifth of the participants (17.0%) were obese. Similarly, more than one in tenth (12.0%) participants had waist circumference categorized as Obese (≥ 102 cm). Also, more than two-fifths (27.7%) of the participants had a waist-to-hip ratio as obese (≥ 1.0).

### Lifestyle Characteristics

More than three-quarters (79.3%) of participants had low (<3000 MET) physical activity. Likewise, nearly two-thirds (65.1%) of the participants did smoking. Among the participants who do smoke, about half (48.5%) of the participants did smoke for longer than seventeen years. Furthermore, more than three-fifths (63.1%) of the participants had alcohol intake. Almost all (98.3%) participants were non-vegetarian. Also, more than three-fifths (63.4%) of the participants had low fruit and vegetable intake (<5 servings). About three-fifths (58.9%) of the participants drive public buses whereas more than half of the participants (55%) had duration of driving for more than thirteen years. Likewise, about three-fifths (57.3%) of the participants had working hours as drivers for more than eight hours. Over a fifth (86.9%) of the participants did rest in between driving. Also, (54.2%) of the participants worked more than forty-eight hours per week and about half of the participants had a weekly average driving of less than 800 km.

### Prevalence of Hypertension

This study found that the prevalence of hypertension among public vehicle drivers was 41.9%, with 64 (18%) being newly diagnosed and 86 (24%) having been previously diagnosed. Additionally, 25 drivers (7%) had never had their blood pressure checked, and half of this group had been identified with hypertension. Furthermore, only 28 drivers (8.7%) were taking the antihypertensive medicine as prescribed, although 37 drivers (11.1%) had prescriptions for it from their doctors.

### Factors associated with Hypertension

Table 2 shows the crude and adjusted odds ratios for factors associated with hypertension. After adjustment, age [adjusted odds ratio (aOR)=1.84, 95%CI 1.13,3.00], BMI (aOR=0.46, 95%CI 0.27,0.76), alcohol (aOR 0.44, 95%CI 0.26, 0.73), and Medication (aOR=0.05, 95%CI 0.01-0.48|aOR 0.05, 95% CI 0.01-0.22).

According to this study, people 35 years of age or older had 1.84 times the odds of having hypertension compared to people under that age (aOR: 1.84, 95% CI: 1.13–3.00, p = 0.013). However, among those with more education (≥10 years), the odds decreased to 0.49, suggesting a significant preventative effect (aOR: 0.49, 95% CI: 0.25–0.98, p = 0.044). A BMI of 25 or higher was associated with a lower chance of developing hypertension (aOR: 0.46, 95% CI: 0.27–0.76, p = 0.003), and alcohol consumption was also found to be protective against hypertension, with users showing lower risks (aOR: 0.44, 95% CI: 0.26–0.73, p = 0.002). Medication status, however, indicated a significant association with the illness, although limited physical activity (p = 0.441) and a family history of hypertension (p = 0.168) did not.

## Discussion

We found less than half of drivers had hypertension, which was associated with age, substance use, and positive family history. The prevalence of hypertension was similar to at least one other study in the region, conducted in India (41.3%)^31^. However, this prevalence is above levels typically reported for other professionals in this occupation. Examples include long-distance truck drivers in south-west Nigeria (39.7%),^28^ among bus drivers in Ghana (38.7%)^32^, bus drivers in Colombo Sri Lanka(36.7%)^33^, professional drivers in Spain(34.7%)^34^, and bus drivers in India (23.8-34.8%)^27,35^. We found only two studies with a higher prevalence of hypertension, namely, public transport drivers in India (49.9%)^36^ and professional drivers in Iran (54.2%)^37^. The observed variances may be attributed to differences in operational definitions, sample sizes, and measurement instruments. The possible explanation for the variation in the prevalence of hypertension may be due to obesity and low physical activity, which could contribute to the increasing prevalence of hypertension.

Our data also revealed age as a determinant of hypertension. A similar study conducted in Ethiopia^38^, Nagpur India^27^, Cameroon^39^, and Ghana^32^ coincides with the findings of this study age of the driver was a higher risk of hypertension. The risk of cardiovascular diseases, particularly hypertension, increases with age. As people age, their body systems change, including the cardiovascular system, leading to cell degeneration that increases the risk of cardiovascular conditions such as hypertension.^40^ These results imply that while addressing the risks of hypertension in occupational groups such as drivers, age is an essential demographic component to observe.

Moreover, our study found a significant association between alcohol use and hypertension. While alcohol intake is generally considered a risk factor for hypertension, our findings suggest a protective association(aOR: 0.44,95% CI:0.26,0.73, p=0.002) that is different from those studies conducted in Ethiopia^38^ south-west Nigeria ^28^ and various occupational groups in Nepal^24^. However, our findings suggest a protective association (aOR: 0.44, 95% CI: 0.26–0.73, p = 0.002) that is different from those reported in studies conducted in Ethiopia^37^, southwestern Nigeria^27^, and Nepal^23^ in other occupational groups. This contrasting result could come from drivers’ apprehension to reveal their alcohol consumption to researchers.

Additionally, our findings revealed a significant association between hypertension and Body Mass Index (BMI). This aligns with research conducted in India Nagpur^27^ in Nigeria^28^ in South Africa^41^ and truck drivers in Ethiopia^42^. Drivers with a BMI of ≥25 exhibited a higher prevalence of hypertension, highlighting the necessity for workplace interventions aimed at weight management. Encouraging physical activity and healthy food practices may reduce this risk among public vehicle drivers. Alarmingly, our study showed that 71.9% of participants reported low physical activity levels. Previous studies indicate that Ethiopian drivers who engage in regular exercise are less prone to developing hypertension.^38^, reinforcing the need for lifestyle modifications.

Furthermore, Higher education (≥10 years) showed a protective impact against hypertension (aOR: 0.49, 95% CI: 0.25–0.98, p = 0.044), indicating that persons with higher educational levels might have enhanced health awareness and access to preventive resources. Medication status was strongly associated with hypertension, with diagnosed but untreated patients having considerably decreased chances (aOR: 0.02, 95% confidence interval: 0.01-0.22, p = 0.001). This may be due to differences in drivers’ self-reported hypertension awareness and medication adherence.

We recognize two major limitations of our study. First, the study employed convenience sampling, potentially constraining the generalizability of the results to the entire population of public vehicle drivers in Pokhara. This approach may create selection bias, since drivers who were readily accessible and eager to participate may not accurately represent the larger community of drivers. Second, since the research was conducted using a cross-sectional design, we were unable to establish the direction of causality. Nonetheless, it is unlikely that men with hypertension and risk factors for hypertension tend to become drivers.

## Conclusions

Study found less than half of drivers had hypertension, which was associated with age, alcohol and substance use, and family history of hypertension. Drivers with these particular risk factors should be targeted for screening and management of hypertension. The research emphasizes the need to focus on modifiable risk factors smoking, alcohol and substance consumption, obese and duration of working hours per day/week, and duration of service.

## Figures and Tables

**Table 1:** Demographic and Lifestyle-related Characteristics of Public Vehicle Drivers in Pokhara, Nepal

Variables	No. (%)
**Age**	
Early adulthood (<35)	147 (41.1)
Middle adulthood (≥35)	211 (58.9)
**Ethnicity**	
Relatively advantage	96 (26.8)
Relatively disadvantage	262 (73.2)
**Years of schooling**	
<10 years	209 (58.4)
≥ 10 years	149 (41.6)
**Marital status**	
Married	332 (92.7)
Unmarried	26 (7.3)
**Income (NRs)**	
≤ 17300	12 (3.4)
> 17300	346 (96.6)
**Family history of HTN**	
Yes	137 (38.3)
No	221 (61.7)
**Waist**	
Normal (<94 cm)	237 (66.2)
Obese (≥ 94 cm)	121 (33.6)
**Waist-hip ratio**	
Normal (<0.9)	311 (86.9)
Obese (≥ 0.9)	47 (13.1)
**Body mass index**	
< 25	132 (36.9)
≥ 25	226 (63.1)
**Physical activity**	
Low (<3000 MET)	284 (79.3)
Adequate (≥ 3000 MET)	74 (20.7)
**Smoking**	
Yes	224 (62.6)
No	134 (37.4)
**Alcohol**	
Yes	226 (63.1)
No	132 (36.9)
**Diet**	
Vegetarian	6 (1.7)
Non-vegetarian	352 (98.3)
**Fruits and vegetable**	
< 5 serving	228 (63.7)
≥ 5 serving	130 (36.3)
**Type of vehicle**	
Public Bus	211 (58.9)
Others	147 (41.1)
**Duration of driving**	
< 14 years	172 (48)
≥ 14 years	186 (52)
**Working hours per day**	
≤ 8 hours	153 (42.7)
> 8 hours	205 (57.3)
**Work hours per week**	
< 48 hours	164 (45.8)
≥ 48 hours	194 (54.3)
**Weekly average driving**	
<800 km	185 (51.7)
≥ 800km	173 (48.5)

NRS: Nepalese Rupee.

**Table 2:** The prevalence of Hypertension in subgroups and which Factors were Associated with Hypertension of Public Vehicle Drivers in Pokhara, Nepal

Variables	Hypertension (%)	cOR (95%CI)	p-value	aOR (95 %CI)	p-value
Background characteristics					
**Age**					
<35 year	28.6	1		1	
≥35 year	51.2	2.62 (1.67-4.10)	<0.001	**1.84(1.13-3.00)**	0.013
**Educational Status**					
<10 years	49.3	1		1	
≥ 10 years	31.5	0.53(0.29-0.98)	0.043	**0.49(0.25-0.98)**	0.044
**Alcohol consumption**					
Yes	46.2	0.43(0.27-0.68)	<0.001	**0.44(0.26-0.73)**	0.002
No	25	1		1	
**Family history of HTN**					
Yes	51.1	0.54(0.35-0.83)	0.006	0.70(0.43-1.15)	0.168
No	36.2	1		1	
**Body mass index**					
< 25	29.5	1		1	
≥ 25	49.1	0.43(0.27-0.68)	<0.001	**0.46(0.27-0.76)**	0.003
**Physical activity**					
Adequate (≥ 3000 MET)	29.7	1		1	
Low (<3000 MET)	45.1	0.54(0.10-2.86)	0.477	0.42(0.04-3.76)	0.441
**Medication**					
No Diagnosis	44	0.28(0.03-0.24)	<0.001	**0.05(0.01-0.48)**	0.009
Diagnosis but not Treatment	36.5	0.21(0.03-0.15)	<0.001	**0.02(0.01-0.22)**	0.001
On Treatment	96.5	1		1	

## Data Availability

All necessary data supporting the findings of this study are available from the corresponding author upon reasonable request.

## Abbreviations

BP: Blood Pressure
CVD: Cardiovascular Disease
DBP: Diastolic Blood Pressure
ERB: Ethical Review Board
HTN: Hypertension
NCDs: Non- communicable Diseases
OR: Odds Ratio
PE: Physical Exercise
PEN: Package of Essential Non-communicable Diseases
SBP: Systolic Blood Pressure
SEAR: South East Asia Region
SPSS: Statistical Package for the Social Sciences
STEPS: STEP wise Surveillance
WHO: World Health Organization
